# Therapeutic Lymphangiogenesis With Implantation of Adipose‐Derived Regenerative Cells

**DOI:** 10.1161/JAHA.112.000877

**Published:** 2012-08-24

**Authors:** Yuuki Shimizu, Rei Shibata, Satoshi Shintani, Masakazu Ishii, Toyoaki Murohara

**Affiliations:** From the Department of Cardiology, Nagoya University Graduate School of Medicine, Nagoya, Japan

**Keywords:** adipose tissue, adipose‐derived regenerative cells, lymphedema, lymphangiogenesis, vascular endothelial growth factor C, macrophages

## Abstract

**Background:**

Lymphedema is one of the serious clinical problems that can occur after surgical resection of malignant tumors such as breast cancer or intra‐pelvic cancers. However, no effective treatment options exist at present. Here, we report that implantation of adipose‐derived regenerative cells (ADRCs) can induce lymphangiogenesis in a mouse model of reparative lymphedema.

**Methods and Results:**

ADRCs were isolated from C57BL/6J mice. To examine the therapeutic efficacy of ADRC implantation in vivo, we established a new mouse model of tail lymphedema. Lymphedema was improved significantly by local injection of ADRCs (*P*<0.05). Histological analysis revealed that lymphatic capillary density was greater in the ADRC group than in the phosphate‐buffered saline control group (*P*<0.01). Tissue expression of vascular endothelial growth factor C mRNA and plasma levels of vascular endothelial growth factor C were greater in the ADRC group than in the control group (*P*<0.01 and *P*<0.05, respectively). ADRCs released vascular endothelial growth factor C, which directly stimulated lymphangiogenesis. Implantation of ADRCs also enhanced recruitment of bone marrow–derived M2 macrophages, which served as lymphatic endothelial progenitor cells.

**Conclusions:**

Implantation of autologous ADRCs could be a useful treatment option for patients with severe lymphedema via mediation of lymphangiogenesis. **(*J Am Heart Assoc*. 2012;1:e000877 doi: 10.1161/JAHA.112.000877.)**

## Introduction

Lymphedema is caused by an accumulation of excess lymphatic fluid and swelling of subcutaneous tissues due to obstruction, destruction, or hypoplasia of lymphatic vessels.^1^ Secondary lymphedema often occurs in malignant disease of the pelvis or groin and may follow radical surgery or radiation therapy.^1^ Despite substantial advances in surgical strategies for tumors, therapeutic options for secondary lymphedema are very limited, even at present.^2–4^

Recent reports showed that some angiogenic cytokines would augment lymphangiogenesis in animal models of lymphedema.^2–11^ These cytokines include vascular endothelial growth factor (VEGF) C, VEGF‐A, fibroblast growth factor (FGF) 2, angiopoietin‐1, hepatocyte growth factor (HGF), and adorenomedullin.^5–10^ However, the cytokines’ relatively short‐lasting efficacies might hamper the ability of injection of a single cytokine to augment lymphangiogenesis and reduce lymphedema. In fact, similar limited findings have been confirmed in the field of therapeutic angiogenesis, where a placebo‐controlled randomized clinical trial that used a single cytokine, basic FGF (bFGF) gene, failed to show beneficial efficacies in patients with critical limb ischemia (the TAMARIS Study; Efficacy and Safety of XRP0038/NV1FGF in Critical Limb Ischemia Patients With Skin Lesions).^12^

With regard to cell‐based vascular regeneration therapy, we have reported that implantation of autologous bone marrow–derived mononuclear cells (BM‐MNCs) induces angiogenesis in severely ischemic limbs in both basic and clinical studies.^13–15^ A recent report also showed that implantation of bone marrow–derived mesenchymal stem cells induced lymphangiogenesis.^16^ Mesenchymal stem cells usually are collected from bone marrow because it is well known that bone marrow–derived mesenchymal stem cells serve as progenitor (stem) cells for various tissues.^17^ More recently, Zuk and coworkers^18^ demonstrated that adipose tissues contain mesenchymal stem cells termed *adipose‐derived regenerative cells (ADRCs)*, which have an ability to regenerate various tissues. We previously demonstrated that implantation of ADRCs augmented postnatal neovascularization in ischemic tissues by cytokine‐dependent paracrine actions.^19^ Moreover, implanted ADRCs secreted the chemokine stromal‐derived factor–1, which recruited endothelial progenitor cells from bone marrow in vivo.^19^ However, little is known as to whether implantation of ADRCs also could promote lymphangiogenesis in a secondary lymphedema model.

Accordingly, we examined the effects of ADRC implantation on therapeutic lymphangiogenesis in a mouse model of secondary lymphedema. The present study demonstrates that implanted ADRCs release the lymphangiogenic cytokine VEGF‐C, which recruits lymphatic endothelial progenitor cells. By these mechanisms, ADRCs seem to be a useful cell source for therapeutic lymphangiogenesis in patients with severe secondary lymphedema.

## Methods

All protocols were approved by the Institutional Animal Care and Use Committee of Nagoya University School of Medicine. Investigators for the follow‐up examinations were blinded to the identity of animals to which treatment was given.

### Isolation of Mouse ADRCs and BM‐MNCs

Under general anesthesia with pentobarbital sodium (50 mg/kg IP), inguinal fat pads (0.1 to 0.2 g) were isolated from C57BL/6J male mice (20 to 23 g, 7 to 8 weeks old; Nihon Crea, Tokyo, Japan) or green fluorescence protein (GFP)–transgenic mice with C57BL/6J background (kindly provided by Dr M. Okabe at Osaka University, Japan).^20^ Subcutaneous adipose tissues were minced and digested with 2 mg/mL type I collagenase at 37°C (Wako, Japan). After filtration through a 40‐μm‐gauge filter (BD Falcon, Bedford, MA), mature adipocytes and stromal fraction were separated by centrifugation at 1200 rpm for 5 minutes. We used these freshly isolated cells in the pellet as ADRCs.^18,19^

Bone marrow tissues were obtained from the bilateral femurs and tibia of C57BL/6J mice as described previously.^21,22^ BM‐MNCs then were isolated by centrifugation through a Histopaque‐density gradient method as described previously.^21,22^

### Mouse Model of Tail Lymphedema

**Figure 1.**
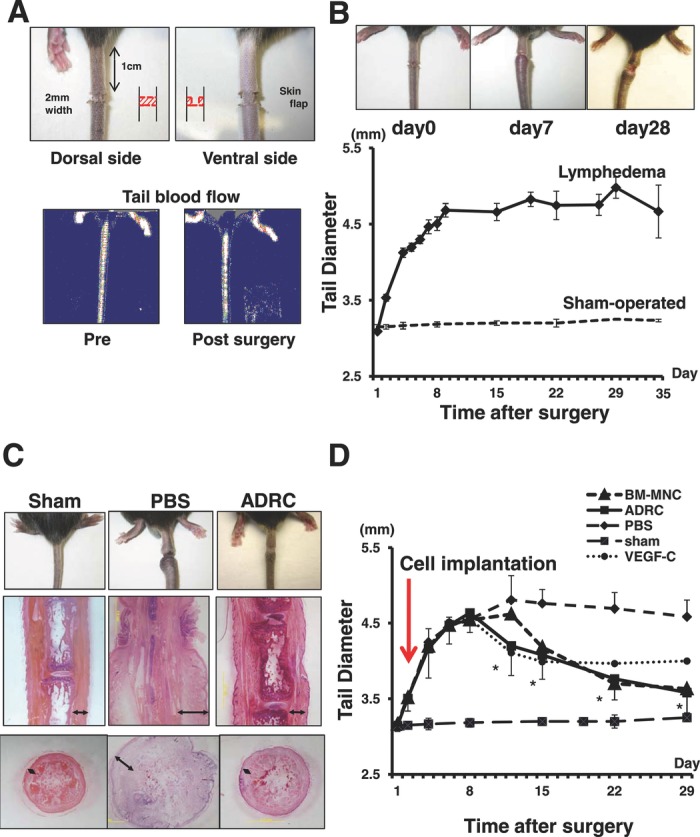
Newly established mouse model of tail lymphedema. A, Circumferential annulus of skin (2 mm wide) located 10 mm distal to the tail base, excluding a 4‐mm^2^ dermal flap at the ventral side, was removed from the tail. Good blood perfusion was maintained at distal site. B, Tail lymphedema was induced within a few days after the procedure, and the tail diameter peaked at around postoperative day 7. Lymphedema continued in the PBS group to at least 28 days. C, Representative photomicrographs of histological sections in lymphedematous tail tissues distal to the incision site. The space of subcutaneous tissues was edematous and dilated in the PBS group, but the space was almost normal in the ADRC implantation group (double‐headed arrow). Scale bar=2 mm. D, As a positive control, a marked reduction of the edema was observed after day 11 in VEGF‐C protein–administered group compared to PBS group (*P*<0.05 vs PBS group). Lymphedema was improved by ADRC implantation. Two weeks after cell implantation, a marked reduction of the tail diameter was observed in the ADRC and BM‐MNC groups. *P<0.01 for comparison between the PBS group and ADRC or BM‐MNC groups.

We created a new mouse model of secondary lymphedema after some modifications of previously published methods.^5,11,23^ Male C57BL/6J mice (7 to 8 weeks old, n=36; Crea Japan) were anesthetized with pentobarbital sodium (50 mg/kg IP). Then, a 2‐mm‐wide circumferential annulus of the skin at 10 mm distal to the tail base, excluding a 4‐mm^2^ dermal flap located at the ventral side, was excised from the tail by use of a cautery knife (CHANGE‐A‐TIP Deluxe Low‐Temperature Cautery Kit) (Figure 1A). By this operation, the subcutaneous lymphatic vessels were disturbed. After the surgery, using a laser Doppler blood perfusion image analyzer (Moor Instruments, Devon, UK), we confirmed that blood flow at the distal site of the tail was completely maintained.

### Assessment of Lymphangiogenesis

After surgical induction of tail lymphedema, mice were divided randomly into 3 groups. No mice died during the experimentation. In addition to the sham‐operated group, the control group (n=12) received phosphate‐buffered saline (PBS). The ADRC group (n=12) received freshly isolated ADRCs (2×10^6^ cells per animal) implanted at 2 different points of the lymphedematous skin flap at postoperative day 1. In some experiments, mature adipocytes (2×10^6^ cells per animal) as negative control were implanted at 2 different points of the lymphedematous skin flap at postoperative day 1 (the mature adipocyte group). Freshly isolated BM‐MNCs (2×10^6^ cells per animal) as positive control also were implanted at 2 different points of the lymphedematous skin flap at postoperative day 1. Because VEGF‐C is an established lymphangiogenic growth factor,^24,25^ in some experiments, mice with edematous tails (n=6) received a single subcutaneous injection of recombinant human VEGF‐C (rhVEGF‐C) (4 μg/100 μL per animal) at postoperative day 3, and the degree of tail lymphedema was assessed. Some mice received GFP‐transgenic mice–derived ADRCs for implanted cell trace experiments. After the treatment, the degree of tissue lymphangiogenesis and lymphedema of the tail were analyzed as described below.

### Tail Thickness Measurement

Diameter of the tail simply represents the severity of the lymphedema. Four weeks after the various cell transplantations or rhVEGF‐C treatment, tail diameter at the lymphedematous site was measured at the point exactly 10 mm distal to the incision site. An age‐matched control group of mice was maintained without this surgical procedure (the sham operation group), and the tail diameter was measured at baseline and thereafter.

At the end of the follow‐up, mice were euthanized. Their tails were obtained, sliced, and snap‐frozen with liquid nitrogen. Frozen tissue sections were sliced and stained with hematoxylin‐eosin. To examine the diameter of the lymph vessels distal to the incision site, frozen tissue sections were subjected to immunohistochemical staining with a polyclonal antibody directed against lymphatic vascular endothelial hyaluronan receptor‐1 (LYVE‐1; Acris).

### Migration Assay of ADRCs

Migratory function of neonatal human dermal lymphatic microvascular endothelial cells (HMVEC‐dLy; Lonza) was evaluated by a modified Boyden chamber assay (Transwell, Corning, MA).^25^ HMVEC‐dLy (1×10^5^ cells) with Dulbecco's modified Eagle's medium (DMEM) (Sigma) without serum were placed in the upper chamber of 24‐well trans‐well plates with polycarbonate membrane (8‐mm pores), and DMEM containing rhVEGF‐C protein (R&D Systems) was added at various concentrations (1 or 10 ng/mL), or supernatant from conditioned medium for ADRCs (ADRC‐CM) (after 12 or 24 hours of cell culture) was added to the lower chamber. After incubation for 4 hours, the membrane was washed briefly with PBS, and the upper side of the membrane was wiped gently and fixed with methanol and 4% paraformaldehyde. The membrane then was stained with May‐Grunewald's eosin methylene blue solution (Merck) and Giemsa stain solution (Sigma). Migration of HMVEC‐dLy cells was quantified by counting the migrated cells in 5 randomly selected microscopic fields of the duplicated chambers at a magnification of ×400 for each sample.

### Proliferation and Apoptosis Assay of ADRCs

The proportion of apoptotic HMVEC‐dLy cells after serum starvation was determined by the PreMix WST‐1 cell Proliferation Assay System (TAKARA Bio Inc). In brief, HMVEC‐dLy cells were cultured with 100 μL of DMEM, which did not contain supplement, onto 96‐well plates (3×10^4^ cells in 100 μL of culture medium per well). After 24 hours of serum starvation, the apoptotic cells were incubated with DMEM supplemented with either rhVEGF‐C (1 or 10 ng/mL) or ADRC‐CM obtained at 12 or 24 hours after starting cell culture. Then WST‐1 was added to dishes according to the manufacturer's instructions and incubated for an additional 4 hours, and the absorbance was measured.

### Western Blot Analysis

Endothelial nitric oxide synthase (eNOS) and the extracellular signal‐regulated kinase (Erk) signaling pathway play an important role in amplifying migratory and survival signals of lymphatic endothelial cells (LECs) via the VEGF‐C / VEGF receptor 3 pathway.^25,26^ Thus, we examined the effects of rhVEGF‐C or ADRC‐CM on phosphorylation of eNOS and Erk. Western blot analysis of LEC lysates was performed as described previously.^26^ We cultured LECs for 6 hours with serum‐free DMEM and stimulated them by rhVEGF‐C or ADRC‐CM for 30 minutes.^27^ Rabbit polyclonal anti–phosphorylated Erk (1:1000 dilution; Cell Signaling) or anti–phosphorylated eNOS (1:1000 dilution; Cell Signaling) was used to detect activation status of Erk or eNOS. Membranes were incubated with a 1:1000 dilution of the appropriate horseradish peroxidase–conjugated secondary antibody (GE Healthcare). Enhanced chemiluminescence detection system (GE Healthcare) was used to visualize the immunocomplexes.

### Real‐Time Reverse Transcriptase–Polymerase Chain Reaction Analysis

Total RNA was isolated from cultured ADRCs and from the tissues frozen with liquid nitrogen obtained at day 5 after lymphatic vessel ablation surgery by using TRIzol Reagent (Invitrogen Life Technologies). Real‐time reverse transcriptase–polymerase chain reaction analysis of the podoplanin, VEGF‐C, bFGF, VEGF‐A, HGF, and GAPDH mRNA was performed with 1 μg total RNA on the Mx3000P real‐time reverse transcriptase–polymerase chain reaction system (Stratagene), with SYBR Green I used as a double‐stranded DNA–specific dye, according to manufacturer's instructions (Applied Biosystems). mRNA levels were expressed relative to the levels of GAPDH. The forward primer for podoplanin was as follows: 5′‐ATGTGGACCGTGCCAGTGT‐3′; the reverse primer: 5′‐CGCTCTCTGCGTTGGTA ‐3′. The forward primer for VEGF‐C was as follows: 5′‐ AACGTGTCCAAGAAATCAGCC‐3′; the reverse primer: 5′‐AGTCCTCTCCCGCAGTAATCC‐3′. The forward primer for bFGF was as follows: 5′‐ ACCCACACGTCAAACTAC‐3′; the reverse primer: 5′‐CAGACATTGGAAGAAACAG‐3′. The forward primer for VEGF‐A was as follows: 5′‐ CAGGCTGCTGTAACGATGAA‐3′; the reverse primer: 5′‐AATGCTTTCTCCGCTCTGAA‐3′. The forward primer for HGF was as follows: 5′‐GCCAGGTGACCTTTGCTTTA‐3′; the reverse primer: 5′‐TGAACGTAAAGCCCCTGTTC‐3′. The forward primer for GAPDH was as follows: 5′‐ATGGTGAAGGTCGGTGTG‐3′; the reverse primer: 5′‐ACCAGTGGATGCAGGGAT‐3′.

### Enzyme‐Linked Immunosorbent Assay

Conditioned medium was obtained from cultured ADRCs at 72 hours after the final change of fresh DMEM with 10% FBS. Concentrations of VEGF‐C proteins in the media were determined by a mouse VEGF‐C enzyme‐linked immunosorbent assay kit (Cusabio Biotech Co, Ltd) according to the manufacturer's instructions. Plasma levels of VEGF‐C at day 5 after the tail lymphedema surgery were also measured in the sham operation group, the control group, and the ADRC group (n=8 in each group).^19^

### Flow Cytometry

A total of 5×10^5^ BM‐MNCs were incubated for 30 minutes at 4°C with monoclonal antibody directed against LYVE‐1 (Acris) and CD11b (BD Biosciences). To analyze the proportion of LYVE‐1^+^CD11b^+^ double‐positive cells, fluorescence‐activated cell sorter analysis was performed with the FACS Caliber instrument (Becton Dickinson) and Cell Quest software (BD Biosciences).

### Bone Marrow–Derived CD11b^+^ Macrophage Kinetics Assay

We examined whether implanted ADRCs could survive and secrete VEGF‐C for augmentation of lymphangiogenesis and whether M2 macrophages were mobilized from bone marrow. At postoperative day 28, we euthanized mice to harvest the tail tissue. Frozen sections were stained with anti–VEGF‐C (Santa Cruz Biotechnology, Inc), anti–LYVE‐1 (Acris), anti‐CD11b (SRT AbD), or anti‐CD163 (Santa Cruz Biotechnology, Inc), and the nuclei were stained with 4′,6‐diamidino‐2‐phenylindole (DAPI).^28–30^

### Statistics

Results are expressed as means ± standard errors of the mean. Statistical significance was determined with unpaired Student *t* test for comparison between the 2 groups and with 1‐way ANOVA for comparison among ≥3 groups, followed by the Tukey procedure for pairwise comparisons. We also performed repeated ANOVA to assess changes over time, followed by the Tukey procedure for pairwise comparisons. *P* values <0.05 denoted statistical significance.

## Results

### Establishment of a New Mouse Model of Tail Lymphedema

We have established a new mouse model of tail lymphedema without disturbance of blood flow (Figure 1A). In this model, special care was taken to maintain the integrity of blood vessels and tendons so that the tail distal to the skin incision site did not become ischemic or necrotic. In fact, after the surgical procedure, maintenance of blood perfusion was confirmed in the distal tail via laser Doppler blood flowmetry (Figure 1A). At postoperative day 7, lymphedema was stably formed, and it was maintained without necrosis until day 28 (Figure 1B). Consistent with previous reports,^24^ the lymphedema was significantly improved by rhVEGF‐C treatment (a positive control), as judged by the diameter of the edematous tail (Figure 1D).

### ADRC Implantation Reduces Lymphedema

**Figure 2.**
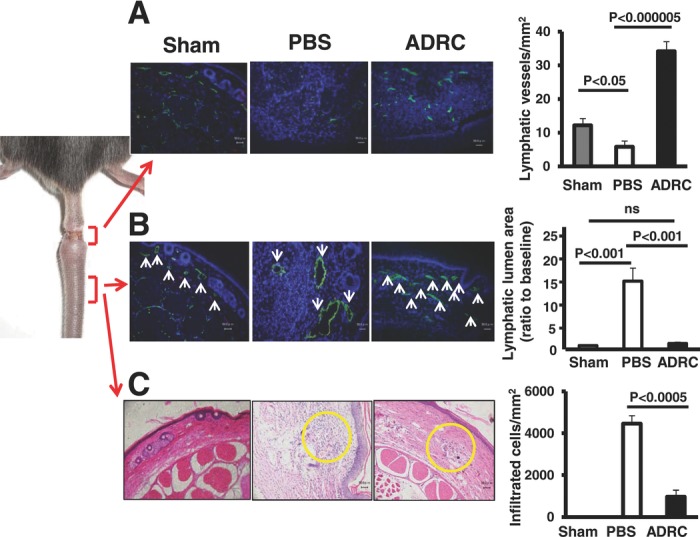
Lymphangiogenesis, edema, necrosis, and secondary infection. A, Immunohistochemical fluorescence staining for LYVE‐1 (green) revealed the presence of numerous capillary LECs in an ADRC‐implanted animal. However, few capillary LECs were observed in a control animal. Scale bar=50 μm. Quantitative data suggest significantly greater capillary density in the ADRC group than in the control group (n=5 for each group). B, Representative fluorescence microscopy of cross sections of tails stained with LYVE‐1 (green) and DAPI (blue). ADRC implantation normalized the size of dilated lymph vessels (white arrowheads). Scale bar=50 μm. Quantitative analysis revealed that the lymphatic lumen area at the distal site of incision was significantly greater in the control group than in the other 2 groups. C, Although there are many infiltrated cells at the lymphedema site in the control group, a lower number of inflammatory cells was seen in the ADRC group. Bar=50 μm. Quantitative analysis revealed that the number of infiltrated cells at tail tissues was significantly lower in the ADRC group than in the control group.

We examined whether implantation of ADRCs could reduce tail lymphedema. After postoperative day 14, a marked reduction of tail lymphedema was observed in ADRC‐implanted mice (n=12), as well as in BM‐MNC–implanted mice (n=12) and rhVEGF‐C–treated mice (n=6) (2 positive control groups), but not in the control PBS‐treated group (n=9) (Figure 1C and 1D). Hematoxylin‐eosin staining of histological sections revealed markedly enlarged tail and edematous interstitial space, with leukocyte infiltration in subcutaneous tissues, in the PBS‐treated control animals. On the other hand, the interstitial space was not enlarged and the numbers of infiltrated cells were reduced in the ADRC‐treated animals (Figure 2C). In addition, typical “elephantiasis”‐like dermal hyperplasia, known as one of the complications of lymphedema, was observed frequently in the PBS control group at day 28, but this was not detected in mice of the ADRC group.^31,32^ Although the diameter of lymphatic vessels distal to the skin incision was dilated markedly in the PBS control group (ie, congestion of lymphatic fluid), the diameter was not dilated in the ADRC implantation group (Figure 2B), which suggests good drainage of lymphatic fluid in this group.

### ADRC Implantation Accelerates Lymphangiogenesis at Congestive Lymphedema Region

**Figure 3.**
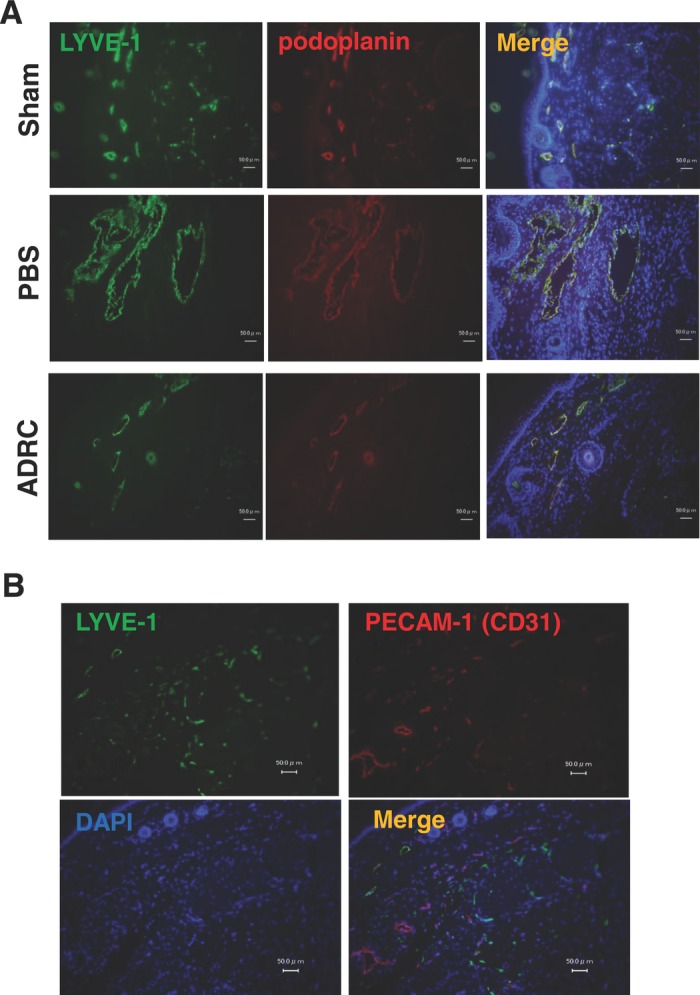
Immunofluorescence staining revealed that lymphatic endothelium was detected by LYVE‐1 and podoplanin double‐positive cells (A) but not by platelet endothelial cell adhesion molecule (PECAM)–1–positive cells in the edematous tissue (B).

Newly formed lymphatic vessels can drain lymphatic fluid from edematous tissues to the venous circulation. In other words, lymphangiogenesis is an important means to prevent lymphedema. We thus examined whether implantation of ADRCs could induce lymphangiogenesis on site. Representative fluorescence immunohistochemical images of LYVE‐1 immunostaining are shown in Figure 2A. Quantitative analysis revealed that the lymphatic vessel density at the cell‐injected region was significantly greater in the ADRC group than in the PBS control group. Furthermore, anti‐podoplanin antibody was used as another marker to determine lymphatic endothelium. Immunofluorescence staining revealed that LYVE‐1–positive cells coexpressed podoplanin but not platelet endothelial cell adhesion molecule‐1 in the edematous tissue (Figure 3A).

**Figure 4.**
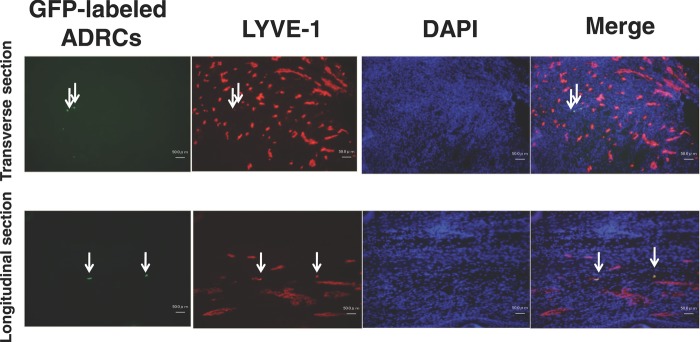
Only a few implanted cells differentiated into LECs in vivo. Four weeks after implantation of GFP mice–derived ADRCs (green), fluorescence microscopic examination of frozen sections from lymphedematous tissues revealed that few GFP‐positive cells were found in implanted area (white arrows) and that these cells seemed to be incorporated into new lymphatic vessels (LYVE‐1 staining; red); however, most of the LECs constructing lymph vessels were not positive for anti–GFP–fluorescein isothiocyanate. Scale bar=50 μm.

We next explored potential mechanisms of the ADRC implantation–enhanced lymphangiogenesis. First, we examined whether implanted ADRCs could directly differentiate into LECs and participate in lymphangiogenesis in vivo. For this purpose, we used GFP‐transgenic mice. Four weeks after implantation of GFP‐transgenic mice–derived ADRCs, fluorescence microscopic examination of frozen sections from lymphedema tissues showed that only a few GFP‐positive cells were found in the implanted area (GFP‐positive cells: 0.5±0.3% per field) (Figure 4).

### Lymphangiogenic Cytokine Production by ADRCs

**Figure 5.**
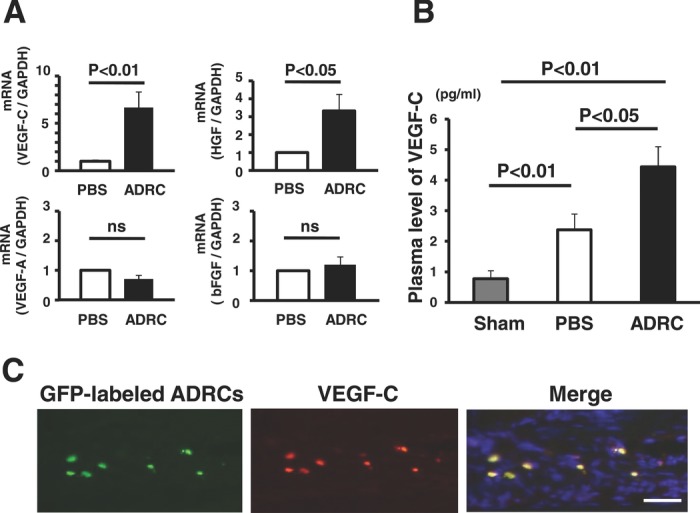
Implantation of ADRCs and lymphangiogenic cytokines in vivo. A, The abundance of VEGF‐C and HGF mRNA in the ADRC group was significantly greater than that of the control group by real‐time reverse transcriptase–polymerase chain reaction (VEGF‐C: 6.7‐fold, n=5, *P*<0.01; HGF: 3.3‐fold, n=5, *P*<0.05). There was no statistically significant difference with regard to the expression of VEGF‐A and bFGF mRNA between the 2 groups. B, Plasma concentration of VEGF‐C in the PBS group was significantly higher than that of sham operation group. VEGF‐C concentration was markedly elevated in the ADRC group compared to the PBS group. C, Implanted GFP‐transgenic mice–derived ADRCs were stained with anti–VEGF‐C mAbs. Bar=50 μm.

We examined the expression of several angiogenic/lymphangiogenic cytokines in the tissues after ADRC transplantation. Although the expression levels of VEGF‐A and bFGF mRNA in the lymphedematous tissue were not significantly different between the ADRC group and the control group, VEGF‐C and HGF mRNA abundance was significantly greater in the ADRC group than in the control group (Figure 5A). We then hypothesized that the main mechanism of the ADRC implantation–enhanced lymphangiogenesis might be paracrine effects of implanted ADRCs. We next performed an enzyme‐linked immunosorbent assay in circulating peripheral blood. In control animals receiving PBS, the plasma level of VEGF‐C was elevated significantly at postoperative day 5 as compared to the baseline values. At day 5, the plasma VEGF‐C level was significantly greater in the ADRC group than in the PBS group (Figure 5B). To further confirm whether ADRCs secrete VEGF‐C protein in situ, frozen sections from lymphedematous tissues of mice that received GFP‐transgenic mice–derived ADRCs were stained with an anti–VEGF‐C mAb. We found that these implanted GFP‐positive ADRCs were costained positive for VEGF‐C in the lymphedematous tissues (Figure 5C), which suggests that implanted ADRCs release VEGF‐C protein.

### Conditioned Media From ADRCs Augments Migration and Proliferation of LECs

**Figure 6.**
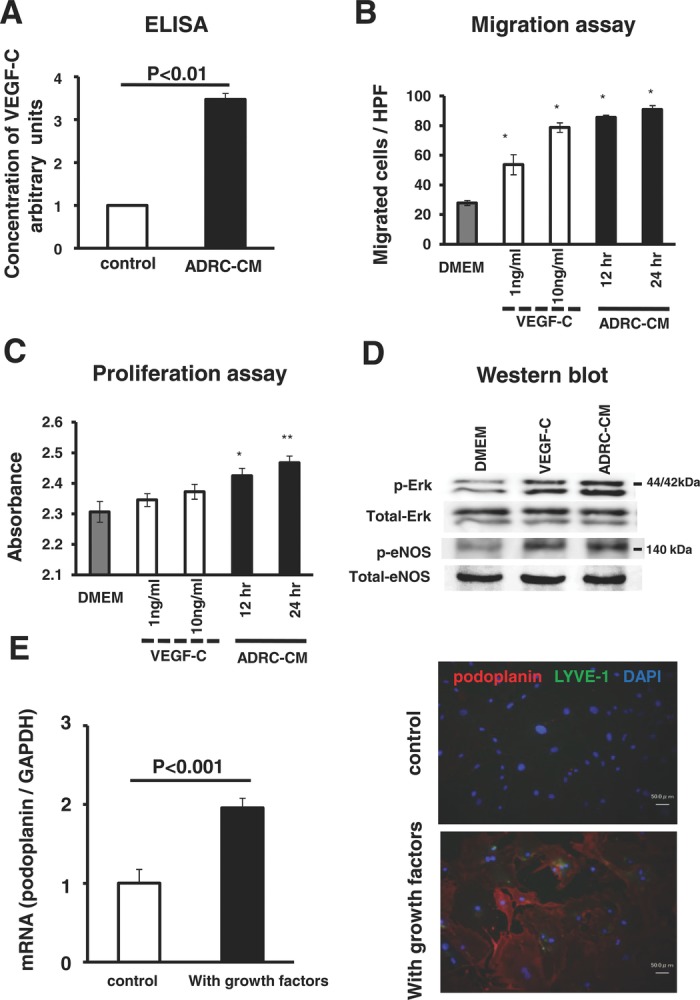
The ability of ADRCs for lymphangiogenesis in vitro (functional assay and differentiation assay). A, Enzyme‐linked immunosorbent assay (ELISA) revealed that the concentration of VEGF‐C was upregulated in ADRC‐CM compared to control. B, rhVEGF‐C induced LEC migration in a dose‐dependent manner, and ADRC‐CM also induced LEC migration. HPF indicates high‐powered field. *P<0.05 vs nontreated control cells. C, Proliferation assay also revealed that ADRC‐CM promoted LEC proliferation, but VEGF‐C did not. *P<0.05, **P<0.01 vs control. D, Western blot analysis revealed that ADRC‐CM, as well as VEGF‐C, had an ability to phosphorylate Erk and eNOS in LECs. Western blot analysis showed that phosphorylated Erk (p‐Erk) and phosphorylated eNOS (p‐eNOS) expression was greater in the VEGF‐C– or ADRC‐CM–treated LECs than in control nontreated LECs. E, Cultured with growth factors, the expression of podoplanin mRNA was greater than that of control. Immunocytochemistry also revealed that cultured ADRCs were stained with LEC marker.

To assess whether ADRCs secrete VEGF‐C protein in vitro, we performed an enzyme‐linked immunosorbent assay in ADRC‐CM. Concentrations of VEGF‐C of conditioned medium from ADRCs were significantly greater than in controls (Figure 6A).

ADRC‐CM enhanced migration of HMVEC‐dLy cells in the modified Boyden chamber assay in vitro. Similarly, rhVEGF‐C, as a positive control, augmented HMVEC‐dLy migration (Figure 6B). The cell proliferation assay also revealed that ADRC‐CM promoted proliferation of HMVEC‐dLy cells; however, rhVEGF‐C did not stimulate the proliferation (Figure 6C). Western blot analysis revealed that phosphorylation of Erk and eNOS, which have been shown to play important roles in suppressing LEC apoptosis, was enhanced in LECs treated with VEGF‐C or ADRC‐CM but not in untreated control LECs (Figure 6D). These results further suggest that cultured ADRCs release cytokines in vitro that stimulated LEC migration and proliferation.

Furthermore, we assessed whether ADRCs could differentiate into LECs in vitro. ADRCs were cultured with medium containing EGM‐2MV. Real‐time polymerase chain reaction and immunofluorescence analysis revealed that lymphatic endothelium cells were selectively induced from ADRCs (Figure 6E).

### Bone Marrow–Derived M2 Macrophages Might Participate in Lymphangiogenesis as LEC Progenitors

**Figure 7.**
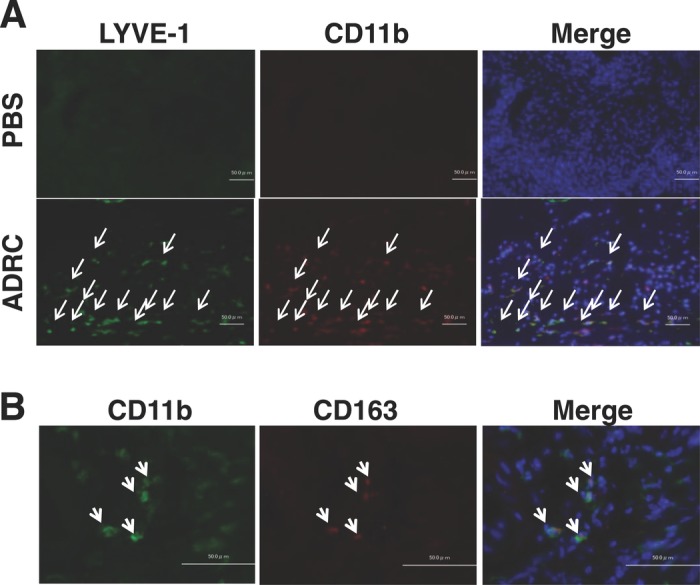
Bone marrow–derived M2 macrophages participate in lymphangiogenesis. A, Immunofluorescence staining revealed that many of the LYVE‐1–positive cells (green) were costained with anti‐CD11b (red). Bar=50 μm. B, Most of these cells were also positive for CD163 (red), an M2 macrophage marker. Bar=50 μm.

**Figure 8.**
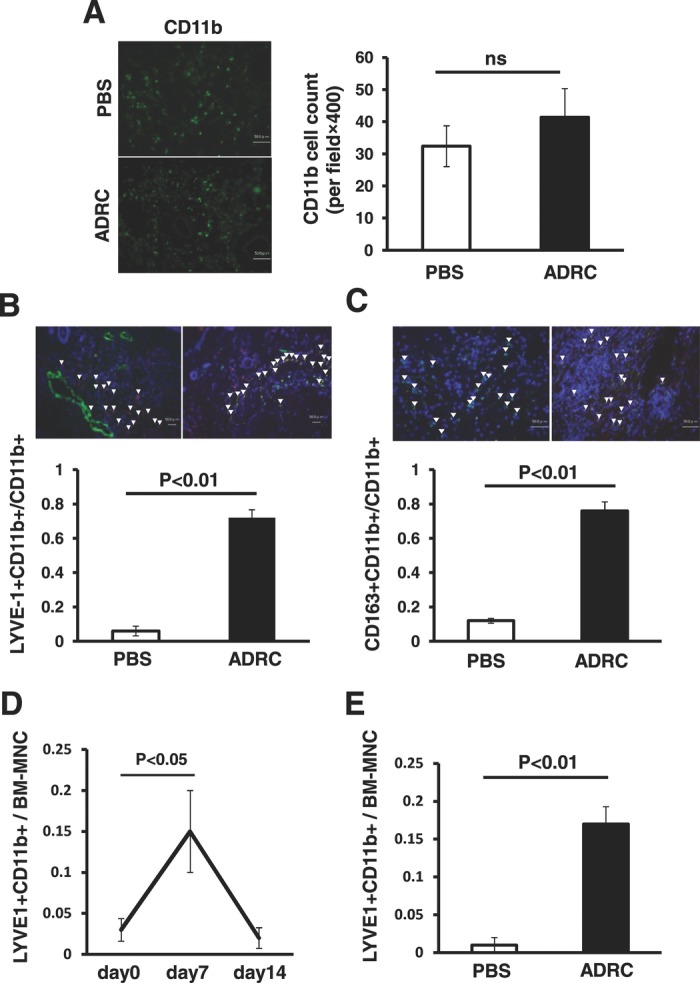
Implantation of ADRCs augments mobilization and/or recruitment of bone marrow–derived M2 macrophages serving as lymphatic endothelial progenitors. A, There were no significant differences in the number of CD11b‐positive cells between the ADRC group and the PBS group after surgery at local tissue. B, Percentage of LYVE‐1–positive cells to CD11b‐positive cells (LEC progenitors) at local tissue was significantly greater in the ADRC group than in the PBS group. C, These cells were also positive for CD163. D, The proportion of LYVE‐1/CD11b double‐positive cells in bone marrow was increased in response to lymphedema. E, The magnitude of this induction was greater in the ADRC‐treated mice than in the control group at day 14.

**Figure 9.**
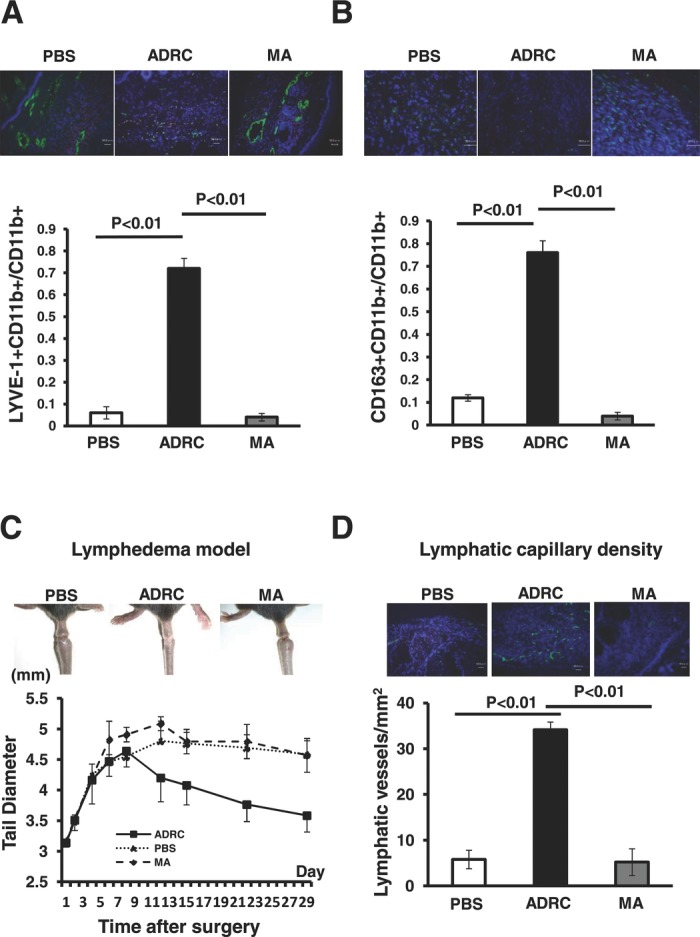
Mature adipocytes (MA) also were administered as a control cell line. Implantation of mature adipocytes did not affect the recruitment of LYVE‐1–positive M2 macrophages (A and B) or the promotion of lymphangiogenesis (C and D).

We further explored additional mechanisms. As mentioned above, direct local implantation of ADRCs into edematous tissues significantly augmented lymphangiogenesis. Immunofluorescence staining revealed that some of the LYVE‐1–positive cells were costained with anti‐CD11b mAb, a macrophage marker (Figure 7A). In addition, most of the CD11b‐positive cells were costained with anti‐CD163 mAb, an M2 macrophage marker (Figure 7B). There were no significant differences in the proportion of CD11b‐positive cells between the ADRC group and the PBS group after surgery at local tissue (Figure 8A). However, quantitative analysis revealed that the percentage of LYVE‐1–positive cells to CD11b‐positive cells was significantly greater in the ADRC group than in the PBS and mature adipocyte groups, which suggests that more LECs developed. These cells were also positive for CD163, a marker of M2 macrophage (Figure 8B through 8C and Figure 9A and 9B). In addition, the proportion of LYVE‐1^+^CD11b^+^ double‐positive cells in bone marrow was increased in response to lymphedema. The magnitude of this induction was much greater in the ADRC group than in the PBS group (Figure 8D and 8E). These data suggest that ADRC implantation might recruit M2 macrophages and that some of these cells might serve as lymphatic endothelial progenitor cells. In other words, newly formed lymphatic vessels initiated by ADRC implantation possibly were caused not only by paracrine factor–mediated residual lymphangiogenesis but also by mobilization and recruitment of bone marrow–derived LEC progenitor–like M2 macrophages. Because CD163‐positive cells were costained with an anti–LYVE‐1 mAb, M2 macrophages seemed to serve as lymphatic endothelial progenitor cells. On the other hand, implantation of mature adipocytes did not promote lymphangiogenesis (Figure 9C and 9D).

## Discussion

**Figure 10.**
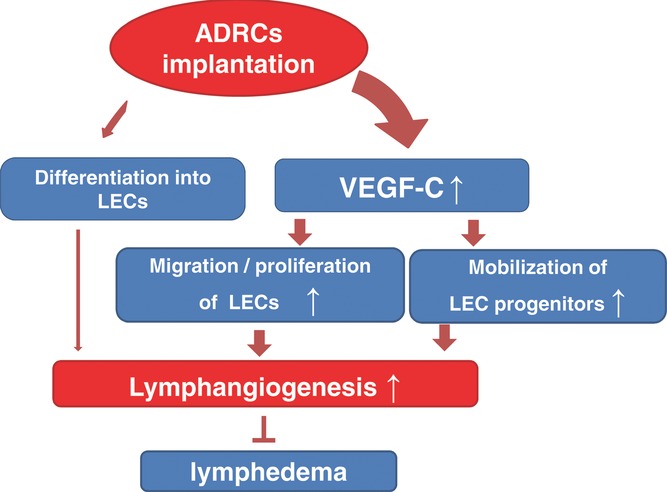
Possible mechanisms of lymphangiogenesis mediated by ADRC implantation. We propose 2 main mechanisms: First, implanted ADRCs release cytokines, including VEGF‐C, that might stimulate migration and proliferation of residual LECs and eventual lymphangiogenesis. Second, cytokines released from ADRCs could augment mobilization and/or recruitment of bone marrow–derived M2 macrophages serving as lymphatic endothelial progenitors. There is little evidence that implanted ADRCs directly transdifferentiate into mature LECs.

Major findings of the present study are that: (1) Direct local implantation of ADRCs elicited lymphangiogenesis in a mouse model of tail lymphedema. (2) Cultured ADRCs released VEGF‐C, which stimulated lymphangiogenesis. (3) ADRC implantation increased VEGF‐C release from lymphedematous tissues in vivo. (4) ADRC implantation enhanced recruitment of bone marrow–derived M2 macrophages, which served as possible lymphatic endothelial progenitor cells. By these multiple mechanisms, implanted ADRCs might have stimulated lymphangiogenesis in a model of reparative lymphedema (Figure 10).

Most of the secondary lymphedema in humans results from a destruction of lymphatic vessels by radical surgery or radiotherapy for malignant tumors. Because there is a paucity of therapeutic options so far, it is necessary to develop an efficacious treatment method for secondary lymphedema.^2,3,4,33^ In the present study, we provide several new findings with regard to therapeutic lymphangiogenesis by ADRC implantation. First, implantation of ADRCs effectively augmented lymphangiogenesis by releasing a cytokine, VEGF‐C. Second, ADRC implantation recruited M2 macrophages, which served as lymphatic endothelial progenitor cells. Third, these results suggest that implantation of autologous graft‐versus‐host disease–free ADRCs will be an important therapeutic option for patients with severe secondary lymphedema. Fourth, we used freshly isolated ADRCs in this study, whereas 2 previous research groups assessed the effects of cultured adipose‐derived cells on lymphangiogenesis.^34,35^

Previously, it was reported that a single administration of a cytokine such as recombinant VEGF‐C protein stimulated lymphangiogenesis in animal models.^24^ However, it was limited by its short‐term efficacy and need for repeated treatments. In contrast, implantation of ADRCs might stably and continuously secrete multiple lymphangiogenic cytokines, including VEGF‐C, from the implanted cells and host cells.

ADRCs not only differentiate into mesenchymal tissues but also can secrete multiple angiogenic growth factors, such as VEGF and HGF.^36,37,38^ We previously found that implantation of ADRCs induced angiogenesis not by a direct differentiation into endothelial cells but by releasing cytokines, including stromal‐derived factor–1, in a mouse model of hind‐limb ischemia.^19^ In the present study, we found that ADRCs released VEGF‐C in vitro and that implantation of ADRCs enhanced VEGF‐C levels in the implanted tissues and peripheral circulation in vivo. Thus, major mechanisms of ADRC transplantation on lymphangiogenesis are most likely mediated through the cytokines/chemokines released, such as VEGF‐C, rather than by a direct differentiation of transplanted ADRCs into lymphatic endothelium.

VEGF‐C that acts via binding to VEGF receptor 3 has been identified as one of the first and most potent lymphangiogenic factors.^39^ Indeed, overexpression of the VEGF‐C gene increased lymphatic vessel density and lymphatic hyperplasia in the skin or in tumors and increased the rate of lymphatic metastasis as a result of abnormal formation and enlargement of peritumor lymphatic vessels.^40^ Taken together, VEGF‐C acts as a key mediator for the lymphangiogenic actions of implanted ADRCs.

It is well established that nitric oxide derived from the endothelial eNOS molecule is beneficial for vascular protection and prevents atherosclerosis development.^41,42^ We previously demonstrated that eNOS regulates endothelial cell migration and angiogenesis in the setting of tissue ischemia.^43,44^ More recently, it has been reported that eNOS mediates VEGF‐C–induced lymphangiogenesis.^26^ VEGF‐C indeed activated eNOS in LECs, and that nitric oxide donor induced proliferation or survival of cultured LECs.^26^ Furthermore, ablation of eNOS activity by either genetic or pharmacological methods abolished regeneration of lymphatic vessels.^26^ In the present study, conditioned media obtained from ADRC cultivation significantly stimulated the phosphorylation of eNOS in LECs. Taken together, ADRC‐induced lymphangiogenesis might be mediated at least in part through the VEGF‐C/VEGF receptor 3 / eNOS activation pathway.

There is another interesting new finding in the present study. Macrophages play a pivotal role in the establishment of the chronic inflammatory state.^27,45,46^ Polarization of mononuclear phagocytes into the M1 “classically activated” macrophages or the M2 “alternatively activated” macrophages has been believed to be a decisive factor in various pathological conditions. M2 macrophages contribute to inflammatory angiogenesis and to the tumor cell's invasion.^47,48^ Interestingly, we found that ADRC implantation enhanced recruitment of bone marrow–derived M2 macrophages that were stained positive for LYVE‐1, a lymphatic endothelial marker. Therefore, some of the M2 macrophages possibly served as lymphatic endothelial progenitor cells. Thus, the M2 macrophage regulatory properties may account for a part of the lymphangiogenesis mediated by ADRCs in the present study.

Recently, it was reported that macrophages apparently perform 2 different roles in lymphangiogenesis.^45^ Macrophages serve as a source of VEGF‐C production and trigger sprouting of preexisting lymphatic vessels and thereby a growth of lymphatic vessels. Alternatively, macrophages transdifferentiate into LECs.^46^ In addition, LYVE‐1^+^ macrophages are found in adipose tissue.^49^ It is possible that LYVE‐1 macrophages in adipose tissue could contribute to lymphangiogenesis. Thus, detailed biochemical studies will be required to elucidate the precise role of (M2) macrophages in lymphangiogenesis.

### Clinical Implications

Although tissue lymphedema is one of the serious clinical problems that can occur after resection of malignant tumors such as breast cancer or intra‐pelvic cancers, there are no effective treatment options so far. Our present study clearly demonstrated that implantation of ADRCs at the site of lymphatic edema induced lymphangiogenesis and reduced tissue edema. Compared to bone marrow aspiration, subcutaneous adipose tissues can be obtained by less invasive methods such as

liposuction, and autologous ADRCs can be separated on site and do not induce any immunologic adverse reaction. Thus, our present findings open a new window for possible therapeutic lymphangiogenesis in patients with critical lymphedema.

In conclusion, implantation of ADRCs accelerates lymphangiogenesis in a congestive lymphedema region in mice. Clearly, additional investigations in larger animal species are warranted, but our present method might provide a novel strategy for therapeutic lymphangiogenesis for patients with severe lymphedema in the near future.
